# Changes in economic activity and mental distress among young adults during the COVID-19 pandemic: Differences between the first and second infection waves in the UK

**DOI:** 10.1371/journal.pone.0292540

**Published:** 2023-10-18

**Authors:** Linruo Zhang, Thierry Gagné, Anne McMunn

**Affiliations:** ESRC International Centre for Life Course Studies in Society & Health, Research Department of Epidemiology and Public Health, University College London, London, United Kingdom; Singapore General Hospital, SINGAPORE

## Abstract

**Background:**

While infection rates, lockdown policies, and labor market conditions substantially varied across COVID-19 waves, the majority of evidence on young adults’ mental health remains focused on initial responses in early 2020. The variability of the relationship between economic activity and mental health over time therefore remains poorly understood in this age group.

**Methods:**

Using linear mixed models, we investigated the relationship between current activity and changes in activity and mental distress (GHQ-12) among 1,390 young adults aged 16–34 via the UK Household Longitudinal Study COVID-19 survey. The association was explored in the first (from April to July 2020) and second (from September 2020 to March 2021) infection waves. Current activity was defined as “not working”, “working <17.5 hours/week”, “17.5–35 hours/week”, and “> = 35 hours/week”. Changes in activity were derived from current and pre-pandemic working hours and divided into four categories: “working with no reduced hours”, “working fewer hours”, “no longer working”, and “did not work before the pandemic”.

**Results:**

During the first wave, no association reached statistical significance. During the second wave: 1) compared to “currently not working”, working 35 or more hours was associated with decreased distress (*b* = -1.54; 95%CI -2.39, -0.69) and working less than 17.5 hours was not (*b* = -0.62; 95%CI -1.66, 0.41); 2) compared to “working with no reduced hours compared with before the outbreak”, no longer working was associated with increased distress (*b* = 1.58, 95%CI 0.61, 2.55) and working with reduced hours was not (*b* = 0.47, 95%CI -0.24, 1.17).

**Conclusion:**

Above the mental health inequalities experienced at the start of the pandemic, full-time work–even with variation in work hours–continued to be a protective factor against mental distress among young adults during the second wave in the UK. Stable, full-time work can better support this age group’s mental well-being over time.

## 1. Introduction

The prevalence of mental health problems among young adults has increased at a rapid pace over the past decade, making this a public health priority [1–4]. Young adults have been experiencing increasingly precarious labor market conditions which have likely influenced their mental health over time. In the United Kingdom (UK), the 2008 Great Recession hit young adults particularly hard as they experienced the largest increases in unemployment and the poorest job prospects after the end of the recession compared with other age groups [5]. Whereas unemployment rates slowly fell back to pre-recession levels over the following decade, new jobs made available to young adults have been more precarious in terms of wages, job sector, and stability, leaving them more vulnerable to poor mental health [6]. Evidence from previous generations has demonstrated that these adversities not only affect current wellbeing, but will also have a considerable “scarring” impacts on health and social outcomes (e.g., unemployment spells) in later life [7].

These trends have been made worse by the COVID-19 pandemic, which had a historical impact on the economy and population levels of mental health across the globe [8–10]. In the UK, the first COVID-19 infection wave began in March and peaked in April 2020, and the second infection wave started at the beginning of September and peaked in January 2021 [11]. Labor market conditions dramatically changed during this period, with UK unemployment rates only returning to pre-pandemic levels around the end of 2021 [12].

Compared with older age groups, UK young adults reported increased psychological distress following the first lockdown in April-May 2020 [13–15]. These responses have not been equal, and have hit harder those who were from ethnic minority backgrounds, living in deprived areas, and already financially insecure [16, 17]. Most studies among young adults suggest that population levels of mental distress peaked in the first COVID-19 wave, but never recovered to pre-pandemic levels [18, 19]. In parallel to these changes, young adults have also been more likely to have lost working hours, experienced lower pay, been put on furlough, or have lost their job over the first year of the pandemic compared with older age groups, with people under age 35 accounting for nearly 80% of those unemployed in 2020 [20]. These experiences have also been unequal, further affecting those from ethnic minority groups and recent graduates who did not have enough time to enter the labor market before the pandemic began [20].

Whereas the context in which this new economic downturn occurred differed from previous recessions, these new negative economic experiences are likely to have contributed just as much to the burden of mental health observed in young adults over the past two years [16, 21, 22]. Chandola et al. (2020) found in the UK Household Longitudinal Study that adults reported an increased risk of distress between April and July 2020 if they had lost their employment due to the pandemic, found that their financial situation had become difficult, or expected to be financially worse off in the future [23]. Among young adults, Gagné et al. (2021) found in those aged 16–24 that changes in work since before the outbreak were associated with increased distress between April and November 2020. Ferry et al. (2021) concluded that in UK adults that working fewer hours in April 2020 was not associated with a higher risk of distress, suggesting that work-hour flexibility may have been a successful mitigation strategy in the short term [24]. The COVID-19 pandemic affected people unequally across occupations. Studies found that UK healthcare workers and other key workers reported larger decreases in mental health compared with other people [25, 26]. British gig economy workers also experienced worse mental health compared with full-time and part-time workers (but still better than the unemployed) [27]. The extent to which these associations may have been modified by specific types and patterns of change in economic activity and by the UK Government Coronavirus Job Retention Scheme (i.e., furlough), however, remains unclear [23, 24, 28, 29].

The rapid progression of the COVID-19 pandemic, lockdown measures, and labor market conditions challenges our understanding of the relationship between work and mental health among UK young adults over the past two years, which cannot be fully gleaned from evidence developed around the start of the first COVID-19 wave [1–4, 13, 24, 30, 31]. Negative changes in economic activity may have had different effects on young adults’ capacity to enter or maintain paid work across COVID-19 infection waves [32]. For instance, population levels of stress regarding unemployment, finances, and food insecurity after mid-2020 have varied in line with the progression of the second COVID-19 wave, i.e., peaking around January-February 2021 [19]. Other changes may complicate this relationship: e.g., one qualitative study between June 2020 and January 2021 found that many young adults felt that they had become more open about their mental health and had developed stronger connections with family members as a result of the pandemic, which may act as a new buffer against unemployment in the short term [33].

### 1.1. Objectives

Few studies that we know of have explored how the pandemic has affected the mental health of UK young adults across economic activity groups beyond the first COVID-19 wave [34]. In response, this study aims to estimate the relationship between economic activity (i.e., current activity and potential change compared with before the outbreak) and mental distress over the course of the first and second COVID-19 waves, each capturing different labor market conditions and lockdown strategies. This is done using a longitudinal cohort dataset of young adults aged 16–34 in 2017–18 and followed eight times between April 2020 and March 2021 (see timeline of data collection in S1 and S2 Figs (S1 File)).

## 2. Methods

### 2.1. Data

The UK Household Longitudinal Study (UKHLS) is a large household panel study started in 2009–10 with a clustered and stratified probability sample of households in England, Scotland, and Wales, and a non-clustered, simple random sample of households in Northern Ireland. UKHLS waves are fielded on a two-year basis (e.g., the UKHLS main wave 1 recruited a first general population sample in 2009 and a second sample oversampling ethnic minorities in 2010) and follow participants on a yearly basis.

In April 2020, a UKHLS COVID-19 sub-study was started by contacting all cohort members aged 16+ who participated in the UKHLS main waves 8 (2016–17) or 9 (2017–18). From April to July 2020, cohort members were invited to participate to a survey online every month for a total of four times (C1-C4). As a reference point, response rates for the entire UKHLS sample in the first four web surveys were 42%, 35%, 34%, and 32%, respectively.

From September onwards, the survey was conducted every two months. Only respondents who had at least one partial interview between C1-C4 would be invited to participate in the following surveys. The last three waves C6-C8 also only invited participants who had been invited in the previous wave. Waves C5, C7, and C8 were conducted online in September 2020, January and March 2021 separately. Wave C6 was fielded in November 2020 via both online survey and telephone interview. As a reference point, response rates for the remaining sample in the next four C5-C8 web surveys were 67%, 62%, 63%, and 67%, respectively.

The analytic sample for this study includes the 1,643 cohort members who participated in the UKHLS main wave 9 (2017–18) when they were ages 16–34 and had a valid survey weight in the UKHLS COVID-19 wave C8 (March 2021) (a flow diagram is detailed in S3 Fig (S1 File)). At the time of analysis, having a survey weight at wave C8 required participation in the main wave 9, meaning that those who only participated in the main wave 8 were *de facto* dropped.

### 2.2. Measures

We measured mental distress at each wave (i.e., C1 to C8) with the 12-item General Health Questionnaire (GHQ-12), a rapid and non-invasive screening instrument to identify individuals at risk of developing psychosis in the general population [35]. Another study found that it was a reliable and valid instrument in the UKHLS, with Cronbach’s α ranging from 0.87 to 0.91 across ethnic groups [36]. Participants need to complete twelve items to rate their experiences of mental distress based on a 4-point Likert-type response scale (scored as 0-1-2-3) (items are detailed in S1 Table (S1 File)). Responses are analyzed by summating the 12 items into a score ranging from 0 (least distressed) to 36 (completely distressed). As a reference point to interpret differences in GHQ scores, Pierce et al. (2020) found that GHQ scores in April 2020 had increased on average by 2.7 in those aged 16–24 and 1.6 in those aged 25–34 compared to the 2017–18 baseline [1].

We derived economic activity at each survey wave from employment status and work hours reported at the interview date and retrospectively for January/February 2020 into two measures: 1) current economic activity and 2) changes in economic activity since before the outbreak. Current economic activity was classified into four categories: 1) not working at survey wave; 2) working less than 17.5 hours per week; 3) working from 17.5 to less than 35 hours per week; 4) working 35 or more hours per week. Changes in economic activity were coded into four categories: 1) working in Jan/Feb 2020 part-/full-time, and did not work fewer hours at survey wave; 2) working in Jan/Feb 2020, and worked fewer hours at survey wave; 3) working in Jan/Feb 2020, and no longer worked at survey wave; 4) a residual category representing those did not work in Jan/Feb 2020. We used these two measures to explore different facets of work, i.e., the “current” variable focusing on work hours and the “change” variable focusing on reduced hours and job loss compared with before the pandemic. We note that too few participants worked fewer hours at survey wave compared with Jan/Feb 2020 to distinguish whether they were now working full-time or part-time. Full-time work is defined as working 35 or more hours per week [37].

We included the following variables from the UKHLS main wave 9 (2017–18) as covariates: 1) sex (male/female), 2) age (16-19/20-24/25-29/30-34), 3) ethnicity (White UK/other), 4) long-standing physical or mental illness (yes/no), 5) parenthood (yes/no), 6) marital status (not cohabiting with a partner/cohabiting with a partner/cohabiting and married), 7) employment status (employed or self-employed/unemployed/full-time student/out of the labor force), and 8) mental distress before the pandemic (GHQ-12 continuous 0–36 score). Missingness on the GHQ-12 score and economic activity variables across survey waves is detailed in S7 Table (S1 File).

### 2.3. Statistical analysis

We performed the analyses within the first four waves (C1-C4, April to July 2020) representing the first infection wave and the next four waves (C5-C8, September 2020 to March 2021) representing the second infection wave separately in two steps. First, we present summary descriptive statistics for economic activity and mental distress. Second, we examined how changes in economic activity were associated with mental distress across waves using linear mixed modelling (LMM). LMMs were tested in three main steps for each main predictor, testing: 1) current economic activity or changes in economic activity (both time-varying), with time (i.e., waves C1-C4, and C5-C8) and a random intercept; 2) the same model, now controlling for covariates collected at baseline (UKHLS main wave 9); 3) the same model, now also adding interaction terms between time and the time-varying predictor to test if the magnitude of associations changed within waves C1-C4 and C5-C8 [38]. Finally, we plotted adjusted predicted scores from the interaction models across 16 “categories” (i.e., four activity categories across four time points) to better interpret interactions [39].

Analyses were produced in the complete-case sample of participants with a valid weight, data on economic activity and mental distress in at least one time point, and data on covariates in the UKHLS main wave 9 (i.e., 85% of the analytic sample). Analyses were produced in Stata 17.0 [40]. Clustering and stratification variables provided by the UKHLS team were integrated to account for sampling design [41]. The wave C8 “cross-sectional” weight was applied in all analyses to account for unequal probabilities of sampling and non-response when participants first entered the UKHLS, attrition across the UKHLS main waves, and attrition between the UKHLS main waves 9 and C8. We reproduced the analyses using the UKHLS “longitudinal” weight in the subset of participants who responded in all eight COVID-19 survey waves and found no differences in results.

### 2.4. Ethics statement

The UKHLS is primarily funded by the Economic and Social Research Council (ESRC) and other co-funding organizations [42]. Data collection from the UKHLS main study and its COVID-19 substudy has been systematically approved by the University of Essex Ethics Committee. No additional ethical approval was necessary for this project.

## 3. Results

### 3.1. Sample characteristics

A total of 1,390 participants was included in our sample. Using characteristics from Wave 9 (2017–18), 62.9% were female, 86.1% were White British, 18.6% were cohabiting and married, 13.8% reported having children, 21.9% reported a long-standing physical or mental illness, 65.4% were employed or self-employed, and the mean GHQ-12 score was 11.9. Participants had on average 6.8 valid observations across the eight time points.

Table 1 presents the distribution of economic activity and GHQ-12 scores across the eight waves between April 2020 and March 2021. The distribution of covariates is shown in S2 Table (S1 File). Average GHQ-12 scores of mental distress significantly varied over time: from 14.2 in April 2020, 13.9 in May, 14.1 in June, 13.1 in July, 12.8 in September, 14.1 in November, 14.3 in January 2021, to 13.8 in March 2021. In comparison, the average GHQ-12 score in 2017–18 was 11.9.

**Table 1:** Economic activity and mental distress in the complete-case sample. UKHLS COVID-19, 2020–21 (*n* = 1,390).

	First COVID-19 infection wave				Second COVID-19 infection wave			
	Wave C1 Apr 2020	Wave C2 May 2020	Wave C3 Jun 2020	Wave C4 Jul 2020	Wave C5 Sep 2020	Wave C6 Nov 2020	Wave C7 Jan 2021	Wave C8 Mar 2021
	**%**	**%**	**%**	**%**	**%**	**%**	**%**	**%**
**Current economic activity**								
Not working	49.8	43.3	39.3	35.9	28.0	33.5	32.4	31.7
Working < 17.5 hours per week	6.3	6.9	6.2	7.9	7.4	6.3	7.9	6.7
Working 17.5–35 hours per week	9.2	11.8	12.6	13.6	15.6	12.1	11.9	13.3
Working > = 35 hours per week	34.8	38.0	41.9	42.6	49.0	48.2	47.8	48.3
**Changes in economic activity**								
Worked in Jan/Feb 2020, no fewer hours	39.3	39.0	42.0	43.3	48.3	45.4	42.7	44.5
Worked in Jan/Feb 2020, fewer work hours	9.8	15.1	15.6	17.9	19.0	15.3	18.6	17.8
Worked in Jan/Feb 2020, no longer worked	30.5	24.9	20.2	17.4	10.8	17.1	16.0	16.3
Did not work in Jan/Feb 2020	20.3	21.0	22.2	21.4	21.9	22.2	22.6	21.4
**Mental distress**								
GHQ-12 (range 0–36), mean (SD, SE)	14.2 (10.4)	13.9 (11.4)	14.1 (11.8)	13.1 (10.9)	12.8 (8.6)	14.1 (9.6)	14.3 (9.3)	13.8 (9.4)
	(0.3)	(0.3)	(0.3)	(0.3)	(0.3)	(0.3)	(0.3)	(0.3)

Estimates are weighted. SD = Standard Deviation. SE = Standard error

The size of economic activity groups across time points followed a similar trend. For current activity, the proportion of participants not working declined from a high of 49.8% in April 2020 to a low of 28.0% in September 2020, increased again in November 2020 (33.5%), and remained stable until March 2021 (31.7%). The proportion of participants working 35 or more hours per week similarly increased from 34.8% in April 2020 to 49.0% in September 2020, and then remained stable until March 2021 (48.3%). During this time, the proportion of participants working fewer than 17.5 hours remained relatively stable, ranging from 6.3% in April 2020 to 6.7% in March 2021.

For changes since before the outbreak, the proportion of respondents who worked in January/February 2020 and now worked fewer hours increased from 9.8% in April 2020 to 19.0% in September 2020, and remained stable until March 2021 (17.8%). The proportion of respondents who worked in January/February 2020 and now no longer worked decreased from 30.5% in April 2020 to 10.8% in September 2020, increased again in November 2020 (17.1%), and remained stable until March 2021 (16.3%).

### 3.2. Current activity and mental distress among young adults during the first COVID-19 wave (wave C1-C4)

Table 2 presents results from the models regressing GHQ-12 scores on current economic activity across four waves between April and July 2020 (see detailed results in S3 Table (S1 File)). In the fully-adjusted model, compared to those not working, respondents working 17.5 to <35 hours per week reported a 0.35 lower GHQ-12 score (95% CI -1.26, 0.57; *p* = 0.456) and respondents working > = 35 hours per week reported a 0.68 lower GHQ-12 score (95%CI -1.53, 0.17; *p* = 0.117). The difference for those working less than 17.5 hours per week was also not significant (*b* = -0.21, 95%CI -1.31, 0.88; *p* = 0.699). In Model 3, adding interaction terms, compared to those not working, participants working 35 or more hours per week reported a 1.23 (95%CI -2.24, 0.22, p = 0.017) lower GHQ score in wave C1.

**Table 2:** Mental distress (GHQ) on current economic activity between April 2020 and July 2020 among UK young adults aged 16–34 in 2017–18. UKHLS COVID-19 waves C1-C4, the first COVID infection wave (*N =* 1,387 participants, *n =* 4,914 observations).

	Model 1 before adjustment		Model 2 fully-adjusted		Model 3 with interaction	
	*b*	95%CI	*b*	95%CI	*b*	95%CI
**Exposure (Current economic activity)**						
Not working (ref.)						
Working < 17.5 hours p.w.	-0.37	(-1.45, 0.72)	-0.21	(-1.31, 0.88)	0.04	(-1.56, 1.64)
Working 17.5–35 hours p.w.	-0.55	(-1.45, 0.35)	-0.35	(-1.26, 0.57)	-0.45	(-1.66, 0.75)
Working > = 35 hours p.w.	**-0.94**	**(-1.74, -0.13)**	-0.68	(-1.53, 0.17)	**-1.23**	**(-2.24, -0.22)**
**Time (COVID-19 survey waves)**						
C1 Apr 2020 (ref.)						
C2 May 2020	-0.42	(-0.87, 0.03)	-0.44	(-0.88, 0.01)	**-0.84**	**(-1.55, -0.12)**
C3 Jun 2020	-0.37	(-0.87, 0.13)	-0.40	(-0.90, 0.10)	-0.74	(-1.57, 0.10)
C4 Jul 2020	**-1.19**	**(-1.72, -0.67)**	**-1.22**	**(-1.75, -0.69)**	**-1.47**	**(-2.35, -0.60)**
**Interaction (exposure X time)**						
Working < 17.5h * May 2020					-0.27	(-2.40, 1.87)
Working < 17.5h * Jun 2020					0.25	(-2.02, 2.52)
Working < 17.5h * Jul 2020					-0.65	(-3.13, 1.82)
Working 17.5 – 35h * May 2020					0.31	(-1.06, 1.67)
Working 17.5 – 35h * Jun 2020					0.42	(-1.08, 1.92)
Working 17.5 – 35h * Jul 2020					-0.05	(-1.39, 1.29)
Working > = 35h * May 2020					0.96	(0.03, 1.89)
Working > = 35h * Jun 2020					0.66	(-0.35, 1.67)
Working > = 35h * Jul 2020					0.77	(-0.31, 1.85)

Estimates are betas from weighted mixed linear models with a random intercept using the wave C8 weight. A higher GHQ-12 score indicates higher mental distress. The sample represents respondents with a valid weight value and information on economic activity and mental distress in at least one time point and information on covariates in the UKHLS main wave 9. Models 2 and 3 adjust for sex, age, ethnicity, long-standing physical or mental health condition, parenthood, marital status, employment status, and mental distress before the pandemic (2017–18). CI = Confidence interval.

**Fig 1:**
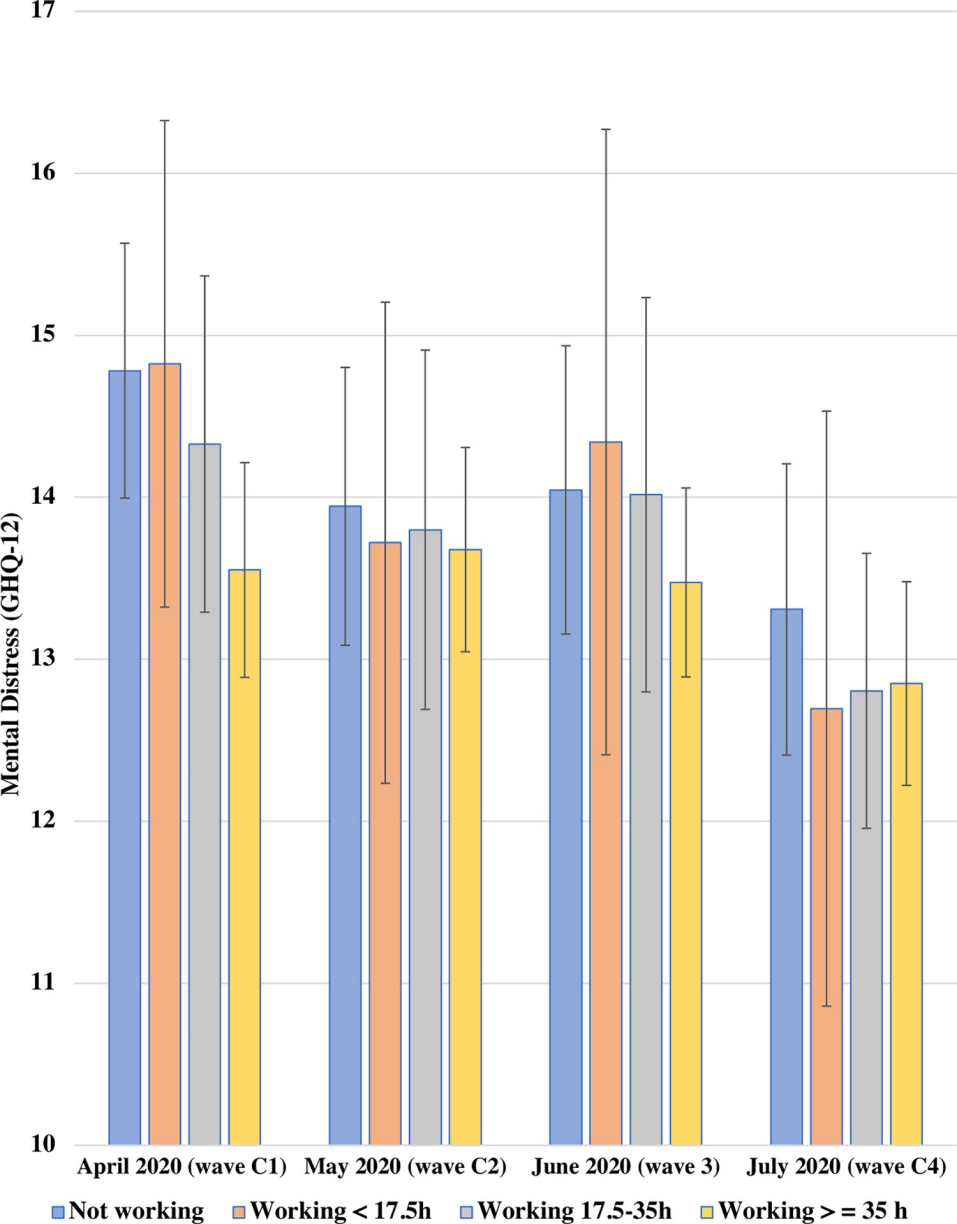
Adjusted predicted GHQ-12 scores between April 2020 and July 2020 by current economic activity among UK young adults aged 16–34 in 2017–18. UKHLS COVID-19 waves C1-C4, the first COVID infection wave (*N =* 1,387 participants, *n =* 4,914 observations). Estimates represent adjusted predicted GHQ-12 scores for current economic activity based on the fully-adjusted model adding the two-way interaction with time. A higher GHQ-12 score indicates higher mental distress.

Fig 1 presents the adjusted predicted GHQ-12 scores estimated from the model with interaction terms (Model 3) across the four current activity groups between April and July 2020. There was no evidence of differences in associations over time when adding the interaction terms (global test *p* = 0.656).

### 3.3. Changes in activity since before the outbreak and mental distress among young adults during the first COVID-19 wave (wave C1-C4)

Table 3 presents results from the models regressing GHQ-12 scores on changes in economic activity since before the outbreak across four waves between April and July 2020 (see detailed results in S4 Table (S1 File)). In the fully-adjusted model, compared to those who worked in January/February 2020 with no fewer work hours at survey wave, those who worked fewer hours reported a non-significant 0.25 higher GHQ-12 score (95% CI -0.42, 0.91; *p* = 0.467) and those who no longer worked reported a 0.48 higher GHQ-12 score (95%CI -0.38, 1.34; *p* = 0.275). In Model 3, adding interaction terms, compared to those who worked in January/February 2020 with no fewer work hours at survey wave, participants who no longer worked reported a 1.08 (95%CI 0.02, 2.14, p = 0.046) higher GHQ score.

**Table 3:** Mental distress (GHQ) on changes in economic activity since before the pandemic between April 2020 and July 2020 among UK young adults aged 16–34 in 2017–18. UKHLS COVID-19 waves C1-C4, the first COVID infection wave (*N =* 1,387 participants, *n =* 4,883 observations).

	Model 1 before adjustment		Model 2 fully-adjusted		Model 3 with interaction	
	*b*	95%CI	*b*	95%CI	*b*	95%CI
**Exposure (Changes in economic activity)**						
Worked in Jan/Feb 2020, no reduced hours (ref.)						
Worked in Jan/Feb 2020, reduced hours	0.31	(-0.39, 1.01)	0.25	(-0.42, 0.91)	1.00	(-0.18, 2.19)
Worked in Jan/Feb 2020, no longer working	0.64	(-0.24, 1.52)	0.48	(-0.38, 1.34)	**1.08**	**(0.02, 2.14)**
Did not work in Jan/Feb 2020	1.22	(-0.01, 2.45)	0.56	(-0.74, 1.86)	1.11	(-0.36, 2.57)
**Time (COVID-19 survey waves)**						
C1 Apr 2020 (ref.)						
C2 May 2020	-0.45	(-0.91, 0.00)	**-0.46**	**(-0.91, -0.01)**	0.14	(-0.51, 0.79)
C3 Jun 2020	-0.44	(-0.94, 0.06)	-0.45	(-0.96, 0.05)	0.04	(-0.61, 0.68)
C4 Jul 2020	**-1.25**	**(-1.78, -0.71)**	**-1.26**	**(-1.80, -0.72)**	**-0.81**	**(-1.52, -0.10)**
**Interaction (exposure X time)**						
Worked in Jan/Feb 2020, reduced hours * May 2020					-1.13	(-2.50, 0.25)
Worked in Jan/Feb 2020, reduced hours * Jun 2020					-1.12	(-2.61, 0.37)
Worked in Jan/Feb 2020, reduced hours * Jul 2020					-0.83	(-2.24, 0.57)
No longer working in Jan/Feb 2020 * May 2020					-1.08	(-2.24, 0.08)
No longer working in Jan/Feb 2020 * Jun 2020					-0.84	(-2.09, 0.41)
No longer working in Jan/Feb 2020 * Jul 2020					-0.77	(-2.19, 0.64)
Did not work in Jan/Feb 2020 * May 2020					-0.88	(-2.13, 0.37)
Did not work in Jan/Feb 2020 * Jun 2020					-0.67	(-1.96, 0.63)
Did not work in Jan/Feb 2020 * Jul 2020					-0.79	(-2.22, 0.64)

Estimates are betas from weighted mixed linear models with a random intercept using the wave C8 weight. A higher GHQ-12 score indicates higher mental distress. The sample represents respondents with a valid weight value and information on economic activity and mental distress in at least one time point and information on covariates in the UKHLS main wave 9. Models 2 and 3 adjust for sex, age, ethnicity, long-standing physical or mental health condition, parenthood, marital status, employment status, and mental distress before the pandemic (2017–18). CI = Confidence interval

**Fig 2:**
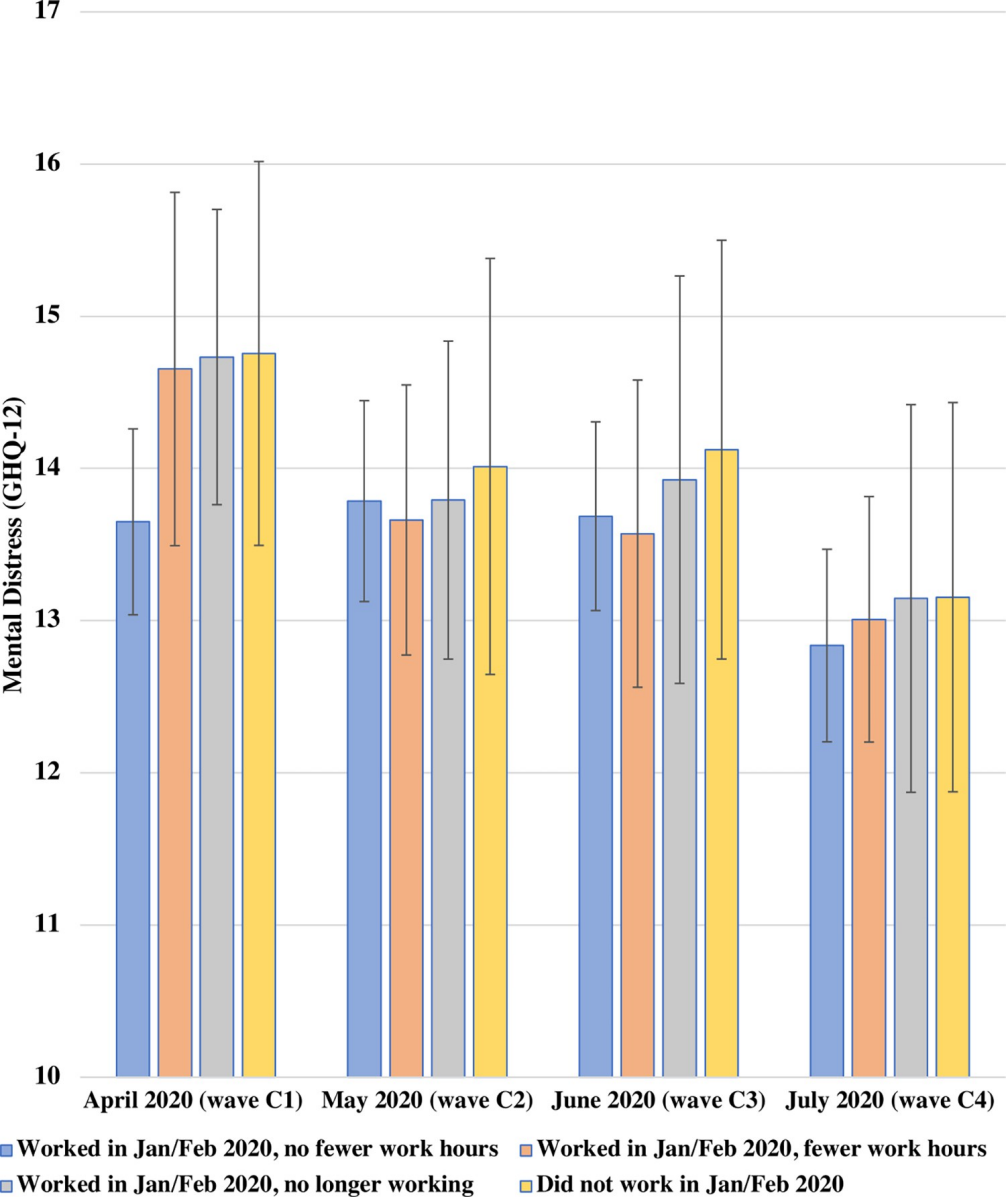
Adjusted predicted GHQ-12 scores between April 2020 and July 2020 by changes in economic activity since before the outbreak among UK young adults aged 16–34 in 2017–18. UKHLS COVID-19 waves C1-C4, the first COVID infection wave (*N =* 1,387 participants, *n =* 4,883 observations). Estimates represent adjusted predicted GHQ-12 scores for changes in economic activity since before the outbreak based on the fully-adjusted model adding the two-way interaction with time and time-varying predictor changes in economic activity since before the outbreak. A higher GHQ-12 score indicates higher mental distress.

Fig 2 presents the adjusted predicted GHQ-12 scores estimated from the model with interaction terms (Model 3) across the four change categories between April and July 2020. There was again no evidence of differences in associations over time when adding the interaction terms (global test *p* = 0.726).

### 3.4. Current activity and mental distress among young adults during the second COVID-19 wave (wave C5-C8)

Table 4 presents results from the models regressing GHQ-12 scores on current economic activity across four waves between September 2020 and March 2021 (see detailed results in S5 Table (S1 File)). In the fully-adjusted model, compared to those not working, respondents working 17.5 to <35 hours per week reported a 0.94 lower GHQ-12 score (95% CI -1.83, -0.05; *p* = 0.038) and respondents working > = 35 hours per week reported a 1.54 lower GHQ-12 score (95%CI -2.39, -0.69; *p* < 0.001). The difference for those working less than 17.5 hours per week, however, remained not significant (*b* = -0.62, 95%CI -1.66, 0.41; *p* = 0.238). In Model 3, adding interaction terms, compared to those not working, participants working 35 hours or more per week reported a 1.41 (95%CI -2.52, -0.30, p = 0.013) lower GHQ score in wave C1.

**Table 4:** Mental distress (GHQ) on current economic activity between Sep 2020 and March 2021 among UK young adults aged 16–34 in 2017–18. UKHLS COVID-19 waves C5-C8, the second COVID infection wave (*N =* 1,390 participants, *n =* 4,502 observations).

	Model 1 before adjustment		Model 2 fully-adjusted		Model 3 with interaction	
	*b*	95%CI	*b*	95%CI	*b*	95%CI
**Exposure (Current economic activity)**						
Not working (ref.)						
Working < 17.5 hours p.w.	-0.69	(-1.70, 0.32)	-0.62	(-1.66, 0.41)	-0.81	(-2.93, 1.31)
Working 17.5–35 hours p.w.	**-1.18**	**(-2.08, -0.28)**	**-0.94**	**(-1.83, -0.05)**	-0.78	(-2.13, 0.57)
Working > = 35 hours p.w.	**-1.79**	**(-2.58, -0.99)**	**-1.54**	**(-2.39, -0.69)**	**-1.41**	**(-2.52, -0.30)**
**Time (COVID-19 survey waves)**						
C5 Sep 2020 (ref.)						
C6 Nov 2020	**1.36**	**(0.82, 1.91)**	**1.37**	**(0.83, 1.90)**	**1.17**	**(0.02, 2.31)**
C7 Jan 2021	**1.46**	**(0.95, 1.98)**	**1.46**	**(0.95, 1.98)**	**1.67**	**(0.72, 2.63)**
C8 Mar 2021	**0.76**	**(0.30, 1.23)**	**0.75**	**(0.29, 1.22)**	0.99	(-0.08, 2.06)
**Interaction (exposure X time)**						
Working < 17.5h * Nov 2020					1.00	(-2.20, 4.21)
Working < 17.5h * Jan 2021					0.19	(-2.73, 3.11)
Working < 17.5h * Mar 2021					-0.09	(-2.70, 2.51)
Working 17.5 – 35h * Nov 2020					0.01	(-1.82, 1.84)
Working 17.5 – 35h * Jan 2021					-0.44	(-2.32, 1.45)
Working 17.5 – 35h * Mar 2021					-0.21	(-2.12, 1.70)
Working > = 35h * Nov 2020					0.27	(-0.96, 1.51)
Working > = 35h * Jan 2021					-0.31	(-1.49, 0.86)
Working > = 35h * Mar 2021					-0.40	(-1.67, 0.88)

Estimates are betas from weighted mixed linear models with a random intercept using the wave C8 weight. A higher GHQ-12 score indicates higher mental distress. The sample represents respondents with a valid weight value and information on economic activity and mental distress in at least one time point and information on covariates in the UKHLS main wave 9. Models 2 and 3 adjust for sex, age, ethnicity, long-standing physical or mental health condition, parenthood, marital status, employment status, and mental distress before the pandemic (2017–18). CI = Confidence interval.

**Fig 3:**
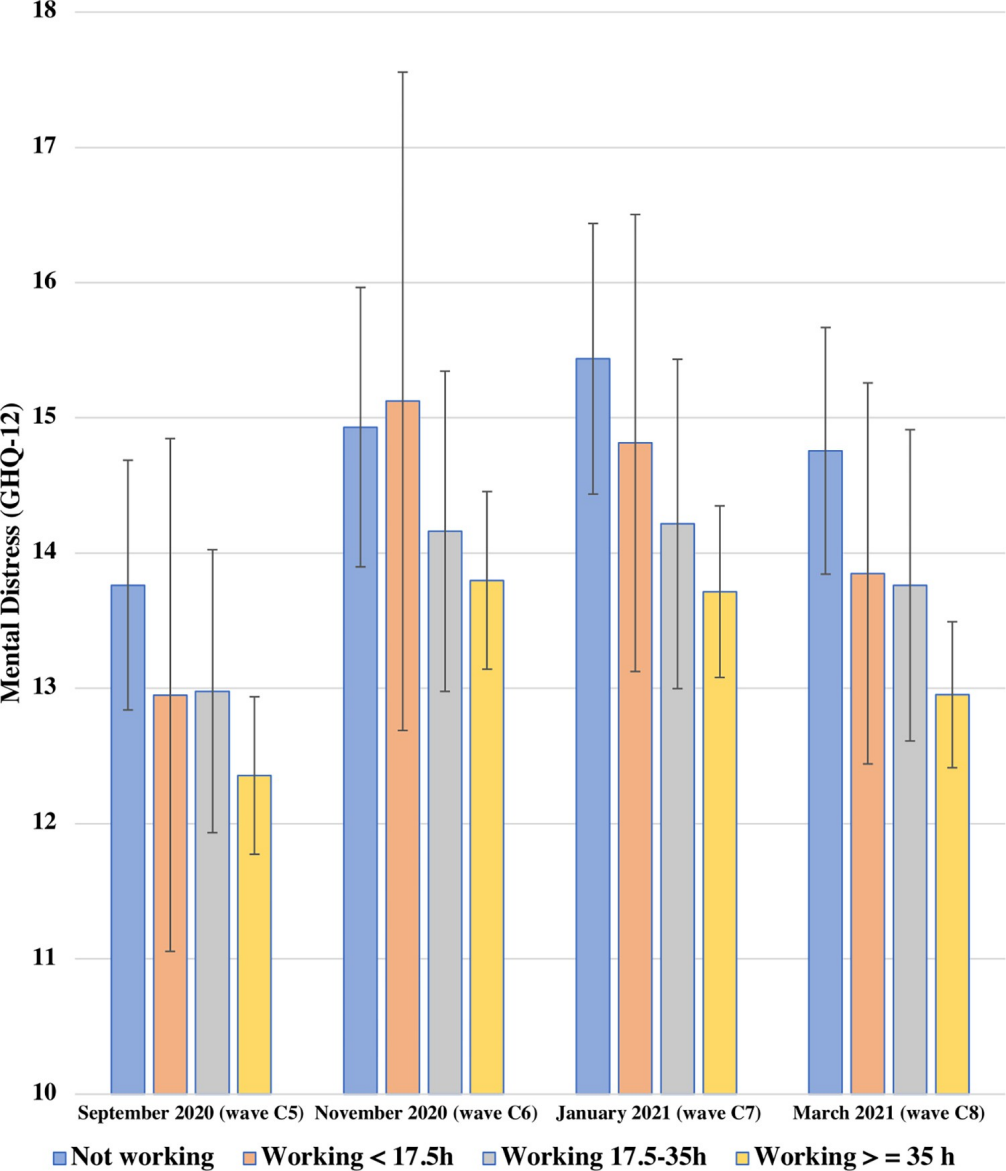
Adjusted predicted GHQ-12 scores between September 2020 and March 2021 by current economic activity among UK young adults aged 16–34 in 2017–18. UKHLS COVID-19 waves C5-C8, the second COVID infection wave (*N =* 1,390 participants, *n =* 4,502 observations). Estimates represent adjusted predicted GHQ-12 scores for current economic activity based on the fully-adjusted model adding the two-way interaction with time and time-varying predictor current economic activity. A higher GHQ-12 score indicates higher mental distress.

Fig 3 presents the adjusted predicted GHQ-12 scores estimated from the model with interaction terms (Model 3) across four current activity groups between September 2020 and March 2021. There was no evidence of differences in associations over time when adding the interaction terms (global test *p* = 0.984).

### 3.5. Changes in economic activity since before the outbreak and mental distress among young adults during the second COVID-19 wave (wave C5-C8)

Table 5 presents results from the models regressing GHQ-12 scores on changes in economic activity since before the outbreak across four waves between September 2020 and March 2021 (see detailed results in S6 Table (S1 File)). In the fully-adjusted model, compared to those who worked in January/February 2020 with no fewer work hours at survey wave, those who worked fewer hours reported a non-significant 0.47 higher GHQ-12 score (95% CI -0.24, 1.17; *p* = 0.193) and those who no longer worked reported a 1.58 higher GHQ-12 score (95%CI 0.61, 2.55; *p* = 0.002). In Model 3, adding interaction terms, compared to those who worked in January/February 2020 with no fewer work hours at survey wave, participants who no longer worked reported a 1.72 (95%CI 0.23, 3.20, p = 0.024) higher GHQ score.

**Table 5:** Mental distress (GHQ) on changes in economic activity since before the pandemic between Sep 2020 and March 2021 among UK young adults aged 16–34 in 2017–18. UKHLS COVID-19 waves C5-C8, the second COVID infection wave (*N =* 1,390 participants, *n =* 4,493 observations).

	Model 1 before adjustment		Model 2 fully-adjusted		Model 3 with interaction	
	*b*	95%CI	*b*	95%CI	*b*	95%CI
**Exposure (Changes in economic activity)**						
Worked in Jan/Feb 2020, no reduced hours (ref.)						
Worked in Jan/Feb 2020, reduced hours	0.65	(-0.08, 1.39)	0.47	(-0.24, 1.17)	0.51	(-0.56, 1.59)
Worked in Jan/Feb 2020, no longer working	**1.85**	**(0.90, 2.81)**	**1.58**	**(0.61, 2.55)**	**1.72**	**(0.23, 3.20)**
Did not work in Jan/Feb 2020	**1.27**	**(0.07, 2.48)**	0.64	(-0.73, 2.01)	0.13	(-1.40, 1.65)
**Time (COVID-19 survey waves)**						
C5 Sep 2020 (ref.)						
C6 Nov 2020	**1.36**	**(0.81, 1.90)**	**1.36**	**(0.83, 1.90)**	**1.45**	**(0.83, 2.07)**
C7 Jan 2021	**1.47**	**(0.96, 1.97)**	**1.47**	**(0.97, 1.97)**	**1.31**	**(0.62, 2.00)**
C8 Mar 2021	**0.75**	**(0.28, 1.22)**	**0.75**	**(0.28, 1.21)**	**0.59**	**(-0.04, 1.22)**
**Interaction (exposure X time)**						
Worked in Jan/Feb 2020, reduced hours * Nov 2020					0.01	(-1.46, 1.48)
Worked in Jan/Feb 2020, reduced hours * Jan 2021					-1.16	(-1.55, 1.22)
Worked in Jan/Feb 2020, reduced hours * Mar 2021					0.00	(-1.45, 1.45)
No longer working in Jan/Feb 2020 * Nov 2020					-0.58	(-2.33, 1.18)
No longer working in Jan/Feb 2020 * Jan 2021					0.17	(-1.57, 1.91)
No longer working in Jan/Feb 2020 * Mar 2021					-0.09	(-1.94, 1.76)
Did not work in Jan/Feb 2020 * Nov 2020					-0.05	(-1.48, 1.39)
Did not work in Jan/Feb 2020 * Jan 2021					0.92	(-0.52, 2.35)
Did not work in Jan/Feb 2020 * Mar 2021					0.94	(-0.35, 2.23)

Estimates are betas from weighted mixed linear models with a random intercept using the wave C8 weight. A higher GHQ-12 score indicates higher mental distress. The sample represents respondents with a valid weight value and information on economic activity and mental distress in at least one time point and information on covariates in the UKHLS main wave 9. Models 2 and 3 adjust for sex, age, ethnicity, long-standing physical or mental health condition, parenthood, marital status, employment status, and mental distress before the pandemic (2017–18). CI = Confidence interval.

**Fig 4:**
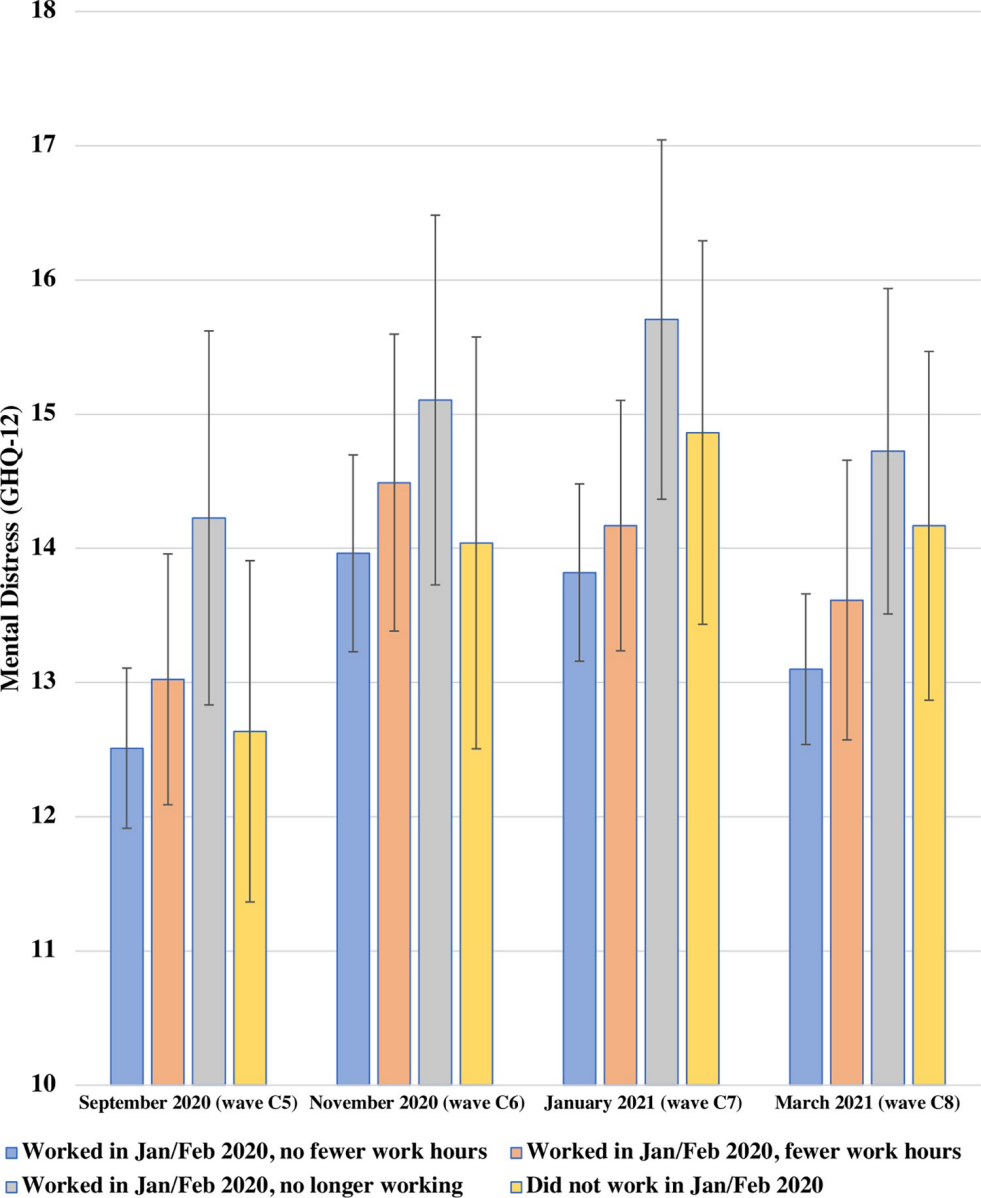
Adjusted predicted GHQ-12 scores between Sep 2020 to March 2021 by changes in economic activity since before the outbreak among UK young adults aged 16–34 in 2017–18. UKHLS COVID-19 waves C5-C8, the second COVID infection wave (*N =* 1,390 participants, *n =* 4,493 observations). Estimates represent adjusted predicted GHQ-12 scores for changes in economic activity since before the outbreak based on the fully-adjusted model adding the two-way interaction with time and time-varying predictor changes in economic activity since before the outbreak. A higher GHQ-12 score indicates higher mental distress.

Fig 4 presents the adjusted predicted GHQ-12 scores estimated from the model with interaction terms (Model 3) across the four change categories between September 2020 and March 2021. There was again no evidence of differences in associations over time when adding the interaction terms (global test *p* = 0.698).

## 4. Discussion

This study explored the variability of the relationship between economic activity and mental distress among UK young adults during the first and second COVID-19 waves, building on the evidence developed at the start of the first COVID-19 wave. We found that, taking into account young adults’ economic activity and mental distress before the pandemic, those who had stopped working reported an increase in mental distress between September 2020 and March 2021. However, this was not the case after the initial shock between April and July 2020. We also found during the second COVID-19 wave that: 1) working part-time (i.e., <17.5 hours per week) was not protective against mental distress compared with not working, and; 2) working fewer hours versus before the outbreak was not associated with a higher risk of mental distress (compared with working similar or more hours). We found no meaningful differences in the magnitude of these associations within the time points used to cover the first or second COVID-19 waves.

The first main finding was that young adults who were not working, or no longer working, reported the highest level of distress during the second COVID-19 wave, which echoes evidence from the start of the pandemic that those who lost their work were one of the most vulnerable groups for mental distress [21, 43, 44]. Beyond financial insecurity, the effects of unemployment on mental health may include boredom, fear of stigma, anxiety about the future, and the loss of time structure, personal agency, and a positive social identity [32, 45]. Some of these effects may have been stronger during the pandemic since social isolation was especially prevalent among young adults who were not working [46].

While working > = 17.5 hours per week was protective against mental distress, working <17.5 hours per week was not during the second wave. In keeping with longstanding changes in labor market conditions over time, differences in mental health between full-time and part-time workers were already increasing over the past decade among UK young adults, with one study finding a 160% larger increase in psychological distress between 2009–10 and 2018–19 in part-time workers compared to full-time workers [16]. The part-time jobs further available to young adults (e.g., sales, manual jobs) have been hit hardest by the pandemic in terms of job security and work scheduling stability, which may have limited any mental health benefits typically associated with part-time work [47]. Part-time workers, also more likely to be women and carers, had a higher risk of having reduced hours and insecure work schedules and a lower chance to regain their initial work hours over time than full-time workers [48]. The furlough scheme is also likely to have been less beneficial for part-time workers compared with full-time workers [48]. Comparing circumstances across infection waves, it is possible that part-time workers had used more of their savings and accumulated more debt by the time the second wave started, leading to more financial distress [48].

Beyond changes which occurred only within the second COVID-19 wave, we also found having continued to work since before the outbreak, even with fewer hours, was protective against mental distress. This is consistent with results by Ferry et al. (2021) who found no association between reduced work hours and changes in mental distress by April 2020 [24]. They also found that this lack of association was robust to the cause of change (e.g., employer-mandated cuts, furlough, caring for others, self-isolation). While reasons for working fewer hours have been extrinsic for most, Ferry et al. proposed that some were able to adapt their work hours with minimal impact (e.g., using annual leave, no longer working overtime). Compared to those who did not reduce their work hours for caring reasons despite wanting to, those who reduced their work hours may have been more likely to use savings and their network’s support [44]. A U.S.-based study also found that for the new group of remote workers, changes in work hours did not always translate into different pay [49]. This supports the idea that work hours captures only one dimension in which work may have changed during the pandemic.

Finally, whereas levels of mental distress substantially varied over the course of the second COVID-19 wave, we did not find evidence that the association between economic activity and mental distress varied within the time points considered. This suggests the effects of unemployment on mental health have been robust to “macro-level” variations, such as changes in worries towards unemployment and financial security, during this time period [19]. Whereas many studies have looked at changes in the relationship between work and mental health across periods of economic change such as the 2008 Great Recession, these have often used longer follow-up periods to highlight differences [5, 50, 51]. It remains to be seen whether the timing of young adults’ economic experiences across COVID-19 waves will have effects on mental health trajectories over time.

### 4.1. Strengths and limitation

This study builds on methodological strengths of the UKHLS, which includes a large sample of young adults who participated before the pandemic and up to eight times over its first year, to derive representative estimates of the role of economic activity in mental distress among young adults during 2020–21. Whereas longitudinal data allowed us to estimate within-person effects, the results based on mixed modelling are still liable to reverse causation and the risk that associations are explained by the omission of confounders not included in the models. Exploring the role of other time-varying factors such as parenthood could also have helped nuance our findings [52]. We highlight two other key limitations. First, response rates in the COVID-19 sub-study waves were low in keeping with the short fieldwork period used to assess responses across multiple, close time points. This led to relatively small sample sizes of young adults, precluding us from considering sex-stratified analyses and limiting our capacity to define meaningful differences as statistically significant. Missing data and low response rates also leave our results more vulnerable to selection bias, which can be only partially limited by including the weights provided by UKHLS. It is possible that young adults who participated were less likely to experience distress from a negative change in economic activity. We explored this by testing differences in employment status and mental distress in the main survey wave 9 (2017–18) between our analytic sample and the initial sample, and found no significant differences. Second, data on furlough status was inconsistently measured across survey waves, meaning that we could not consider it despite being a potentially important factor in nuancing the roles of employment and work hours in mental distress. Besides, given the lower significance of the result found for part-time work, other studies are needed to confirm its protective effect in response to such economic events.

### 4.2. Conclusion

The COVID-19 outbreak has exposed young adults to new challenges, accelerating the worrisome trends which have led this group to a more precarious labor market and deteriorated mental health over the previous decade. Over the course of the second COVID-19 wave in 2020–21, we found that UK young adults who were not working and had lost their job since the start of the pandemic were likely to continue experiencing increases in mental distress. While young adults who worked fewer hours compared with before the outbreak did not report more distress, working part-time was not protective against mental distress during this period. These findings corroborate the arguments that 1) mental health inequalities have continued to increase after the initial shock experienced in early 2020 and 2) *secure*, *full-time employment can better support young adults’ mental well-being in the long run*. Future studies need to confirm whether these findings are robust across work conditions (e.g., industry sector, family arrangements, furlough status), and how these may relate to mental health trajectories in the years to come. Our findings support the need for the UK government to: 1) better understand skills gaps; 2) develop long-term, sustainable infrastructures to tackle them; 3) identify growing job sectors; 4) invest more resources in further education and apprenticeships to support young adults [53].

## Supporting information

S1 File Contains supporting tables and supporting figures. (DOCX)
