# Supplementary material for: Changes in economic activity and mental distress among young adults during the COVID-19 pandemic: Differences between the first and second infection waves in the UK
Source: PLoS One. 2023 Oct 18;18(10):e0292540. doi: 10.1371/journal.pone.0292540 (PMC10584092; doi:10.1371/journal.pone.0292540)
Supplement: S1 File — (DOCX) [file pone.0292540.s001.docx]

**Supporting information**

**Title**

Changes in economic activity and mental distress among young adults during the COVID-19 pandemic: differences between the first and second infection waves in the UK

**Last updated**

May 29^th^, 2023

**Table of content**

- **S1 Table.** GHQ-12 item and response labels.
- **S2 Table.** Distribution of covariates.
- **S3 Table.** C1-C4 Results from fully-adjusted models: current economic activity.
- **S4 Table.** C1-C4 Results from fully-adjusted models: changes since before the outbreak.
- **S5 Table.** C5-C8 Results from fully-adjusted models: current economic activity.
- **S6 Table.** C5-C8 Results from fully-adjusted models: changes since before the outbreak.
- **S7 Table.** Sample size and proportion of missing values.
- **S1 Fig.** Time trends in ILO unemployment rates in the UK.
- **S2 Fig.** Timeline of data collection in the UKHLS COVID-19 substudy.
- **S3 Fig.** Sample flow diagram.

**S1 Table. GHQ-12 items labels**

| **Full items** | |
| --- | --- |
| **1** | **Have you recently been able to concentrate on whatever you're doing?** |
|  | 0. Better than usual 1. Same as usual 2. Less than usual 3. Much less than usual |
| **2** | **Have you recently lost much sleep over worry?** |
|  | 0. Not at all 1. No more than usual 2. Rather more than usual 3. Much more than usual |
| **3** | **Have you recently felt that you were playing a useful part in things?** |
|  | 0. More so than usual 1. Same as usual 2. Less so than usual 3. Much less than usual |
| **4** | **Have you recently felt capable of making decisions about things?** |
|  | 0. More so than usual 1. Same as usual 2. Less so than usual 3. Much less capable |
| **5** | **Have you recently felt constantly under strain?** |
|  | 0. Not at all 1. No more than usual 2. Rather more than usual 3. Much more than usual |
| **6** | **Have you recently felt you couldn't overcome your difficulties?** |
|  | 0. Not at all 1. No more than usual 2. Rather more than usual 3. Much more than usual |
| **7** | **Have you recently been able to enjoy your normal day-to-day activities?** |
|  | 0. More so than usual 1. Same as usual 2. Less so than usual 3. Much less than usual |
| **8** | **Have you recently been able to face up to problems?** |
|  | 0. More so than usual 1. Same as usual 2. Less so than usual 3. Much less than usual |
| **9** | **Have you recently been feeling unhappy or depressed?** |
|  | 0. Not at all 1. No more than usual 2. Rather more than usual 3. Much more than usual |
| **10** | **Have you recently been losing confidence in yourself?** |
|  | 0. Not at all 1. No more than usual 2. Rather more than usual 3. Much more than usual |
| **11** | **Have you recently been thinking of yourself as a worthless person?** |
|  | 0. Not at all 1. No more than usual 2. Rather more than usual 3. Much more than usual |
| **12** | **Have you recently been feeling reasonably happy, all things considered?** |
|  | 0. More so than usual 1. Same as usual 2. Less so than usual 3. Much less than usual |

**S2 Table. Description of time-invariant characteristics (based on measured in UKHLS wave 9 2017-18) in the complete-case sample. UKHLS, 2020-21. (*n* = 1,390).**

| ***At annual UKHLS wave 9*** | |
| --- | --- |
|  | Proportion % |
| **Sex** |  |
| Male | 37.1 |
| Female | 62.9 |
| **Ethnic group** |  |
| White UK | 86.1 |
| Not White UK | 13.9 |
| **Age group** |  |
| 16-19 | 21.8 |
| 20-24 | 26.5 |
| 25-29 | 24.1 |
| 30-34 | 27.6 |
| **Marital status** |  |
| Cohabiting and married | 18.6 |
| Cohabiting and living as couple | 13.6 |
| Not cohabiting | 67.7 |
| **Employment status** |  |
| Employed or self-employed | 65.4 |
| Unemployed | 5.5 |
| Full-time student | 23.4 |
| Out of the labor force | 5.6 |
| **Long-standing physical or mental illness** |  |
| Yes | 21.9 |
| No | 78.1 |
| **Parenthood: having children aged <= 16** |  |
| Yes | 13.8 |
| No | 86.2 |
| **Mental wellbeing at baseline**  GHQ score (range 0-36), mean (SE) | 11.94 (0.30) |

Estimates are weighted. SE = Standard error.

**S3 Table. Mental distress (GHQ) on current economic activity between April and July 2020 among UK young adults aged 16-34 in 2017-18. UKHLS COVID-19 waves C1-C4. (*N =* 1,387 participants*, n =* 4,914 observations)**

|  | **Model 5 fully-adjusted** | |
| --- | --- | --- |
|  | *b* | 95%CI |
| **Exposure (Current economic activity)** |  |  |
| Not working (ref.) |  |  |
| Working with < 17.5 work hours per week | -0.21 | (-1.31, 0.88) |
| Working with 17.5 – 35 work hours per week | -0.35 | (-1.26, 0.57) |
| Working with >= 35 work hours per week | -0.68 | (-1.53, 0.17) |
| **Time (COVID-19 survey waves)** |  |  |
| C1 Apr 2020 (ref.) |  |  |
| C2 May 2020 | -0.44 | (-0.88, 0.01) |
| C3 Jun 2021 | -0.40 | (-0.90, 0.10) |
| C4 Jul 2021 | **-1.22** | **(-1.75, -0.69)** |
| **Sex** |  |  |
| Male (ref.) |  |  |
| Female | 0.79 | (-0.10, 1.68) |
| **Ethnic group** |  |  |
| White UK (ref.) |  |  |
| Not White UK | 0.25 | (-1.00, 1.51) |
| **Age group** |  |  |
| 16-20 (ref.) |  |  |
| 21-25 | 0.02 | (-1.25, 1.28) |
| 26-30 | -0.06 | (-1.65, 1.54) |
| 31-34 | -0.16 | (-1.88, 1.56) |
| **Marital status** |  |  |
| Cohabiting and Married (ref.) |  |  |
| Cohabiting and living as couple | 0.49 | (-0.64, 1.63) |
| Not cohabiting | 0.99 | (-0.20, 2.18) |
| **Employment status** |  |  |
| Employed or self-employed (ref.) |  |  |
| Unemployed | 0.26 | (-1.81, 2.34) |
| Full-time student | -0.31 | (-1.72, 1.09) |
| Out of the labor force | -1.22 | (-2.78, 0.34) |
| **Long-standing physical or mental illness** |  |  |
| No (ref.) |  |  |
| Yes | 0.58 | (-0.39, 1.56) |
| **Parenthood: having children aged <= 16** |  |  |
| No (ref.) |  |  |
| Yes | 0.94 | (-0.38, 2.25) |
| **Mental wellbeing at baseline** | **0.44** | **(0.35, 0.53)** |

**S4 Table. Mental distress (GHQ) on changes in economic activity since before the outbreak between April and July 2020 among UK young adults aged 16-34 in 2017-18. UKHLS COVID-19 waves C1-C4. (*N =* 1,387 participants*, n =* 4,883 observations)**

|  | **Model 2 fully-adjusted** | |
| --- | --- | --- |
|  | *b* | 95%CI |
| **Exposure (Changes in economic activity)** |  |  |
| Worked in Jan/Feb 2020, no reduced hours (ref.) |  |  |
| Worked in Jan/Feb 2020, reduced hours | 0.25 | (-0.42, 0.91) |
| Worked in Jan/Feb 2020, no longer working | 0.48 | (-0.38, 1.34) |
| Did not work in Jan/Feb 2020 | 0.56 | (-0.74, 1.86) |
| **Time (COVID-19 survey waves)** |  |  |
| C1 Apr 2020 (ref.) |  |  |
| C2 May 2020 | **-0.46** | **(****-0.91, -0.01)** |
| C3 Jun 2021 | -0.45 | (-0.96, 0.05) |
| C4 Jul 2021 | **-1.26** | **(****-1.80, -0.72)** |
| **Sex** |  |  |
| Male (ref.) |  |  |
| Female | 0.81 | (-0.09, 1.71) |
| **Ethnic group** |  |  |
| White UK (ref.) |  |  |
| Not White UK | 0.25 | (-1.03, 1.53) |
| **Age group** |  |  |
| 16-20 (ref.) |  |  |
| 21-25 | -0.00 | (-1.28, 1.27) |
| 26-30 | -0.07 | (-1.67, 1.52) |
| 31-34 | -0.22 | (-1.94, 1.51) |
| **Marital status** |  |  |
| Cohabiting and Married (ref.) |  |  |
| Cohabiting and living as couple | 0.47 | (-0.67, 1.61) |
| Not cohabiting | 0.99 | (-0.22, 2.20) |
| **Employment status** |  |  |
| Employed or self-employed (ref.) |  |  |
| Unemployed | 0.29 | (-1.83, 2.41) |
| Full-time student | -0.29 | (-1.74, 1.16) |
| Out of the labor force | -1.21 | (-2.92, 0.50) |
| **Long-standing physical or mental illness** |  |  |
| No (ref.) |  |  |
| Yes | 0.59 | (-0.37, 1.55) |
| **Parenthood: having children aged <= 16** |  |  |
| No (ref.) |  |  |
| Yes | 1.06 | (-0.27, 2.39) |
| **Mental wellbeing at baseline** | **0.44** | **(0.35, 0.53)** |

**S5 Table. Mental distress (GHQ) on current economic activity between September 2020 and March 2021 among UK young adults aged 16-34 in 2017-18. UKHLS COVID-19 waves C5-C8. (*N =* 1,390 participants*, n =* 4,502 observations)**

|  | **Model 5 fully-adjusted** | |
| --- | --- | --- |
|  | *b* | 95%CI |
| **Exposure (Current economic activity)** |  |  |
| Not working (ref.) |  |  |
| Working with < 17.5 work hours per week | -0.62 | (-1.66, 0.41) |
| Working with 17.5 – 35 work hours per week | **-0.94** | **(-1.82, -0.05)** |
| Working with >= 35 work hours per week | **-1.54** | **(-2.39, -0.69)** |
| **Time (COVID-19 survey waves)** |  |  |
| C5 Sep 2020 (ref.) |  |  |
| C6 Nov 2020 | **1.37** | **(0.83, 1.90)** |
| C7 Jan 2021 | **1.46** | **(0.95, 1.98)** |
| C8 Mar 2021 | **0.75** | **(0.29, 1.22)** |
| **Sex** |  |  |
| Male (ref.) |  |  |
| Female | 0.66 | (-0.27, 1.59) |
| **Ethnic group** |  |  |
| White UK (ref.) |  |  |
| Not White UK | 0.09 | (-1.08, 1.25) |
| **Age group** |  |  |
| 16-20 (ref.) |  |  |
| 21-25 | -0.65 | (-1.91, 0.62) |
| 26-30 | -0.77 | (-2.16, 0.60) |
| 31-34 | -0.49 | (-2.05, 1.08) |
| **Marital status** |  |  |
| Cohabiting and Married (ref.) |  |  |
| Cohabiting and living as couple | 0.84 | (-0.47, 2.14) |
| Not cohabiting | 0.97 | (-0.23, 2.18) |
| **Employment status** |  |  |
| Employed or self-employed (ref.) |  |  |
| Unemployed | -0.46 | (-2.23, 1.30) |
| Full-time student | -0.90 | (-2.08, 0.29) |
| Out of the labor force | -1.50 | (-2.90, 0.10) |
| **Long-standing physical or mental illness** |  |  |
| No (ref.) |  |  |
| Yes | 0.58 | (-0.38, 1.55) |
| **Parenthood: having children aged <= 16** |  |  |
| No (ref.) |  |  |
| Yes | 0.35 | (-1.07, 1.78) |
| **Mental wellbeing at baseline** | **0.39** | **(0.30, 0.48)** |

**S6 Table. Mental distress (GHQ) on changes in economic activity since before the outbreak between September 2020 and March 2021 among UK young adults aged 16-34 in 2017-18. UKHLS COVID-19 waves C5-C8. (*N =* 1,390 participants*, n =* 4,493 observations)**

|  | **Model 2 fully-adjusted** | |
| --- | --- | --- |
|  | *b* | 95%CI |
| **Exposure (Changes in economic activity)** |  |  |
| Worked in Jan/Feb 2020, no reduced hours (ref.) |  |  |
| Worked in Jan/Feb 2020, reduced hours | 0.47 | (-0.24, 1.17) |
| Worked in Jan/Feb 2020, no longer working | **1.58** | **(0.61, 2.55)** |
| Did not work in Jan/Feb 2020 | 0.64 | (-0.73, 2.01) |
| **Time (COVID-19 survey waves)** |  |  |
| C5 Sep 2020 (ref.) |  |  |
| C6 Nov 2020 | **1.36** | **(0.83, 1.90)** |
| C7 Jan 2021 | **1.47** | **(0.97, 1.97)** |
| C8 Mar 2021 | **0.75** | **(0.28, 1.21)** |
| **Sex** |  |  |
| Male (ref.) |  |  |
| Female | 0.70 | (-0.22, 1.62) |
| **Ethnic group** |  |  |
| White UK (ref.) |  |  |
| Not White UK | 0.14 | (-1.01, 1.29) |
| **Age group** |  |  |
| 16-20 (ref.) |  |  |
| 21-25 | -0.89 | (-2.16, 0.39) |
| 26-30 | -0.97 | (-2.35, 0.41) |
| 31-34 | -0.67 | (-2.26, 0.92) |
| **Marital status** |  |  |
| Cohabiting and Married (ref.) |  |  |
| Cohabiting and living as couple | 0.84 | (-0.45, 2.13) |
| Not cohabiting | 1.00 | (-0.18, 2.19) |
| **Employment status** |  |  |
| Employed or self-employed (ref.) |  |  |
| Unemployed | -0.15 | (-2.00, 1.70) |
| Full-time student | -0.72 | (-1.99, 0.55) |
| Out of the labor force | -1.11 | (-2.65, 0.42) |
| **Long-standing physical or mental illness** |  |  |
| No (ref.) |  |  |
| Yes | 0.68 | (-0.28, 1.64) |
| **Parenthood: having children aged <= 16** |  |  |
| No (ref.) |  |  |
| Yes | 0.60 | (-0.78, 1.97) |
| **Mental wellbeing at baseline** | **0.39** | **(0.30, 0.48)** |

**S7 Table. Initial observation, valid observation, and proportion of missing values in economic activity or GHQ-12 between April 2020 and March 2021 among UK young adults aged 16-34 in 2017-18. UKHLS COVID-19 waves C1-C8 (*N =* 1,390 participants*, n =* 4,502 observations)*.***

|  | **First COVID-19 infection wave** | | | | **Second COVID-19 infection wave** | | | |
| --- | --- | --- | --- | --- | --- | --- | --- | --- |
|  | **C1 April 2020** | **C2 May 2020** | **C3 Jun 2020** | **C4 Jul 2020** | **C5 Sep 2020** | **C6 Nov 2020** | **C7 Jan 2021** | **C8 Mar 2021** |
| **Initial observation** | 1,451 | 1,321 | 1,279 | 1,275 | 1,200 | 1,134 | 1,150 | 1,554 |
| **Valid observation** | 1,358 | 1,234 | 1,185 | 1,163 | 1,100 | 1,025 | 1,047 | 1,365 |
| **Missing value in economic activity or GHQ-12 (proportion %)** | 6.41% | 6.59% | 7.35% | 8.78% | 8.33% | 9.61% | 8.96% | 12.16% |

**Initial observation** was defined as respondents who participated in UKHLS main wave 9, had a valid weight in COVID-19 wave C8, and had valid covariates.

**Valid observation** was defined as respondents who participated in UKHLS main wave 9, had a valid weight in COVID-19 wave C8, and had valid covariates, economic activity, and GHQ-12.

The proportion of **Missing values in economic activity or GHQ-12 = (initial observation – valid observation) / initial observation.**

**S1 Fig. ILO unemployment rates, ages 16-24 and 25-34. Labour Force Survey, United Kingdom.**

**
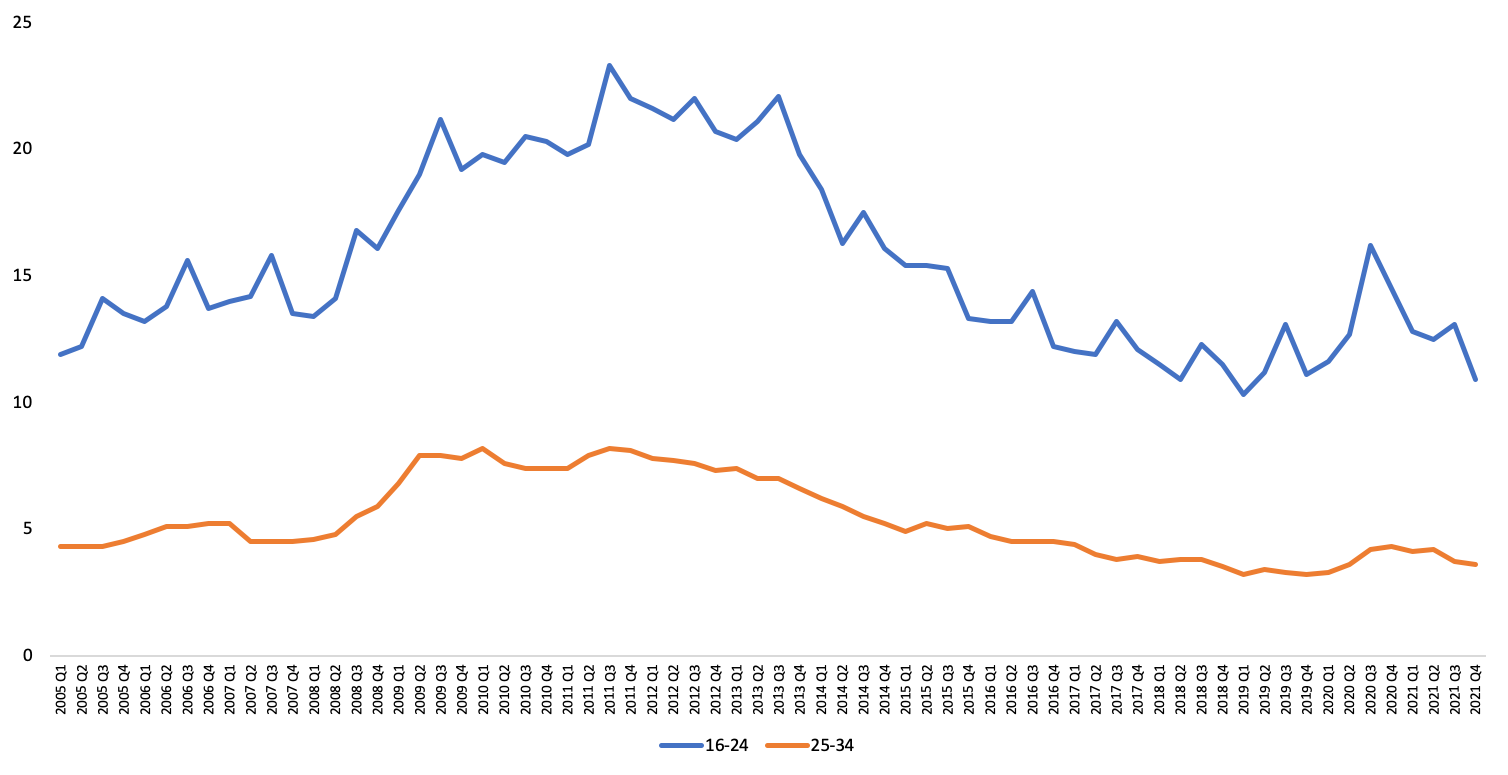
**

**Data is available here (last accessed February 16, 2022):**

1. <https://www.ons.gov.uk/employmentandlabourmarket/peopleinwork/employmentandemployeetypes/timeseries/ycwd/lms>

2. <https://www.ons.gov.uk/employmentandlabourmarket/peoplenotinwork/unemployment/timeseries/ycgp/lms>

**S2 Fig. COVID-19 infection rates and UKHLS COVID-19 study time points.**

**Data is available here (last accessed February 4, 2022):**

<https://coronavirus.data.gov.uk/details/cases?areaType=overview&areaName=United%20Kingdom>

**S3 Fig. Sample flow diagram**

Participants at UKHLS wave 9 aged 16-34, n = 8,996

Ineligible *n* = 7,353

With a valid weight at COVID wave C8, n = 1,643

Ineligible *n* = 89

Valid covariates at UKHLS wave 9, *n* = 1,554

Ineligible *n* = 164

Valid observation in economic activity and GHQ-12 across COVID wave C1-C8, *n* = 1,390

Participate in wave C1, *n* = 1,358

Participate in wave C2, *n* = 1,234

Participate in wave C3, *n* = 1,185

Participate in wave C4, *n* = 1,163

Participate in wave C5, *n* = 1,100

Participate in wave C6, *n* = 1,025

Participate in wave C7, *n* = 1,047

Participate in wave C8, *n* = 1,365
